# Brief interventions for smoking and alcohol associated with the COVID-19 pandemic: a population survey in England

**DOI:** 10.1186/s12889-023-17559-7

**Published:** 2024-01-03

**Authors:** Loren Kock, Lion Shahab, Claire Garnett, Melissa Oldham, Harry Tattan-Birch, Colin Angus, Leonie Brose, Jamie Brown

**Affiliations:** 1 Department of Behavioural Science and Health, University College London, 1-19 Torrington Place, London, WC1E 7HB UK; 2 SPECTRUM Research Consortium, Edinburgh, UK; 3 School of Health and Related Research, The University of Sheffield, Sheffield, UK; 4 Institute of Psychiatry, Psychology and Neuroscience, King’s College London, London, UK

**Keywords:** Brief interventions, Smoking, Alcohol, COVID-19, General practice

## Abstract

**Background:**

Following the onset of the COVID-19 pandemic, in March 2020 health care delivery underwent considerable changes. It is unclear how this may have affected the delivery of Brief Interventions (BIs) for smoking and alcohol. We examined the impact of the COVID-19 pandemic on the receipt of BIs for smoking and alcohol in primary care in England and whether certain priority groups (e.g., less advantaged socioeconomic positions, or a history of a mental health condition) were differentially affected.

**Methods:**

We used nationally representative data from a monthly cross-sectional survey in England between 03/2014 and 06/2022. Monthly trends in the receipt of BIs for smoking and alcohol were examined using generalised additive models among adults who smoked in the past-year (weighted *N* = 31,390) and those using alcohol at increasing and higher risk levels (AUDIT score ^3^8, weighted *N* = 22,386), respectively. Interactions were tested between social grade and the change in slope after the onset of the COVID-19 pandemic, and results reported stratified by social grade. Further logistic regression models assessed whether changes in the of receipt of BIs for smoking and alcohol, respectively, from 12/2016 to 01/2017 and 10/2020 to 06/2022 (or 03/2022 in the case of BIs for alcohol), depended on history of a mental health condition.

**Results:**

The receipt of smoking BIs declined from an average prevalence of 31.8% (95%CI 29.4–35.0) pre-March 2020 to 24.4% (95%CI 23.5–25.4) post-March 2020. The best-fitting model found that after March 2020 there was a 12-month decline before stabilising by June 2022 in social grade ABC1 at a lower level (~ 20%) and rebounding among social grade C2DE (~ 27%). Receipt of BIs for alcohol was low (overall: 4.1%, 95%CI 3.9–4.4) and the prevalence was similar pre- and post-March 2020.

**Conclusions:**

The receipt of BIs for smoking declined following March 2020 but rebounded among priority socioeconomic groups of people who smoked. BIs for alcohol among those who use alcohol at increasing and higher risk levels were low and there was no appreciable change over time. Maintaining higher BI delivery among socioeconomic and mental health priority groups of smokers and increasing and higher risk alcohol users is important to support reductions in smoking and alcohol related inequalities.

**Supplementary Information:**

The online version contains supplementary material available at 10.1186/s12889-023-17559-7.

## Background

The resilience of the health systems around the world – specifically their ability to adapt and minimise the negative consequences of the COVID-19 pandemic on health care delivery – was tested during the COVID-19 pandemic, and important components of preventive health care were disrupted in order to cope with the extra strain caused by the surge in demand for COVID-19 treatment. At the start of the pandemic in England in March 2020, to deal with exponentially growing numbers of seriously ill patients while protecting staff and other patients, certain measures were imposed including redirecting hospital staff and resources, postponement or remote delivery of outpatient services and the suspension of in-person primary care in the community [1]. Even as the initial COVID-19 related restrictions were relaxed, some online and telephone primary care consultations were retained [2]. Prior to the pandemic, there were substantial differences in rates of delivery of brief interventions (BIs) for smoking and alcohol (among those visiting their GP) in England, with 48.3% of people who smoke having received a BI in the past year compared with 6.1% of those who use alcohol at increasing and higher-risk levels [3]. It is important to understand how the COVID-19 pandemic has affected the receipt of BIs for smoking and alcohol, and whether certain priority groups have been differentially affected.

Increasing and higher risk alcohol consumption (a score greater than or equal to eight on the AUDIT (Alcohol Use Disorders Identification Test)) and tobacco smoking remain leading causes of preventable morbidity and mortality [4]. These behaviours are patterned socioeconomically, with the burden falling disproportionately upon certain groups including those with lower incomes or less stable employment [5, 6], and/or with diagnoses of mental health conditions [7, 8]. Socio-economic patterns of alcohol consumption are more complex. Despite people from more disadvantaged backgrounds being more likely to abstain from drinking and drinking less overall than those from more advantaged groups [9], alcohol-related morbidity and mortality is generally higher in this group [10, 11].

National government guidance recommends the use of screening and BIs to combat smoking and increasing and higher risk alcohol use for patients in UK primary care [12–16]. The National Institute for Health and Care Excellence guidelines and a government commissioned report of recommendations to achieve reductions in smoking [17] emphasise the importance of targeting priority groups—often those experiencing multiple disadvantages—who find smoking/alcohol cessation/reduction especially difficult [18]. This emphasis appears to be reflected in evidence of greater receipt of BIs among priority socioeconomic groups in England; however, absolute intervention delivery rates for increasing and higher risk drinkers is low [3, 19].

In the UK, those living with severe mental illness experienced a drop in contact with primary health care after the onset of the pandemic [20]. Changes in healthcare delivery and smoking and drinking behaviour during the ongoing COVID-19 pandemic may have also led to a reduction in the delivery of BIs. If so, it is important to understand whether this has disproportionately impacted those who are at greater risk of harm, such as those experiencing socioeconomic disadvantage or with a history of a mental health condition, particularly as there have been larger increases in the prevalence of increasing and higher risk drinkers among those experiencing socioeconomic disadvantage [21]. Understanding how delivery of BIs for smoking and alcohol in primary care were impacted during the pandemic has relevance in the context of broader health equity given that the groups who were most adversely affected by COVID-19 and the associated response [22] are also those who experience the most harm from smoking and alcohol use.

Using a representative sample of adults in England who smoked in the past year, or used alcohol at increasing and higher risk levels (AUDIT score ≥ 8), this study aimed to examine whether changes in the receipt of BIs for smoking and alcohol (overall and separately excluding those who did not visit their GP in the past year) following the onset of the COVID-19 pandemic compared with previous years and whether receipt was moderated by socioeconomic position (research question 1) or history of a mental health condition (research question 2), respectively.

## Methods

### Design

#### Sample and recruitment

Data were drawn from the Smoking and Alcohol Toolkit Study (STS/ATS), a monthly repeated cross-sectional survey of a representative sample of adults (aged 18 +) in England. The study population consisted of adults aged 18 and over living in households in England surveyed monthly between March 2014 and June 2022. All statistical analysis was restricted to people who smoked in the past year or who used alcohol at increasing and higher risk levels as indicated by scoring ^3^8 in the Alcohol Use Disorders Identification Test (AUDIT) [23].

The STS/ATS uses a hybrid of random location and quota sampling to select a new sample of approximately 1,800 adults (aged ≥ 18 years) each month in England [24]. Sample weighting uses the rim (marginal) weighting technique, an iterative sequence of weighting adjustments whereby separate nationally representative targets are set, and the process repeated until all relevant variables match the English sociodemographic population profile relevant at the time each monthly survey was collected.

Respondents with characteristics that are under-represented receive a larger weight, while those who are over-represented receive a smaller weight. Data were collected monthly through face-to-face computer assisted interviews. However, due to the COVID-19 pandemic, from April 2020 data were collected via telephone only. A series of diagnostic analyses suggested it is reasonable to compare data from before and after the lockdown, despite the change in data collection method [25, 26].

### Measures

#### Outcome

##### *Receipt of a brief intervention for smoking or drinking*

The primary outcome measure was defined using responses to the following questions:

For smoking:

“Has your GP spoken to you about smoking in the past year (i.e. last 12 months)?”

- Yes, he\she suggested that I go to a specialist stop smoking advisor or group
- Yes, he\she suggested that I see a nurse in the practice
- Yes, he\she offered me a prescription for Champix, Zyban, a nicotine patch, nicotine gum or another nicotine product
- Yes, he\she suggested that I use an e-cigarette
- Yes, he\she advised me to stop but did not offer anything
- Yes, he\she asked me about my smoking but did not advise me to stop smoking
- No, I have seen my GP in the last year but he\she has not spoken to me about smoking
- No, I have not seen my GP in the last year
- Don’t know

Respondents who answered with any of responses a-e for smoking were classified as having received a BI. Responses of h were excluded under the sensitivity analyses which cover only those who have visited their GP.

For drinking:

“In the last 12 months, has a doctor or other health worker within your GP surgery discussed your drinking?”

- No
- Yes, a doctor or other health worker within my GP surgery asked about my drinking
- Yes, a doctor or other health worker within my GP surgery offered advice about cutting down on my drinking
- Yes, a doctor or other health worker within my GP surgery offered help or support within the surgery to help me cut down
- Yes, a doctor or other health worker within my GP surgery referred me to an alcohol service or advised me to seek specialist help.
- Don’t know
- Refused

Respondents who answered with any of c-e, were classified as having received a brief intervention from their GP for drinking.

For the analyses including only those who visited their GP, we excluded responses of a) in response to the question below:

“You said a doctor or other health worker within your GP surgery has not discussed your drinking with you in the last 12 months.“

a) I have not seen a doctor or health worker within my GP surgery in last 12 months.

b) I have seen a doctor or health worker within my GP surgery in the last 12 months but did not discuss my drinking.

#### Moderators and covariates

##### *Occupational social grade*

As a measure of socio-economic position, we used the National Readership Survey’s classification of social grade based on occupation (ABC1: higher and intermediate managerial, administrative, and professional, supervisory, clerical and junior managerial, administrative and professional; C2DE: skilled manual workers, semi-skilled and unskilled manual workers and state pensioners, casual and lowest-grade workers, unemployed with state benefits.) [27].

##### *History of a mental health condition*

Respondents were classified as having a history of a mental health condition if they reported being diagnosed by a doctor or health professional.

Respondents were asked:

““Since the age of 16, which of the following, if any, has a doctor or health professional ever told you that you had?”

- Depression
- Anxiety
- Obsessive Compulsive disorder
- Panic disorder or a phobia
- Post-traumatic stress disorder (PTSD)
- Psychosis or schizophrenia
- Personality disorder
- Attention Deficit Hyperactivity Disorder (ADHD)
- An eating disorder
- Alcohol misuse or dependence
- Drug use or dependence
- Problem gambling
- Autism or Autism Spectrum Disorder
- Bipolar disorder (previously known as manic depression)
- None of these
- Don’t know
- Prefer not to say

Responses excluding the final three options above were presented in a randomised order. For our analyses, individual responses of any of the above diagnoses were dummy coded into a composite measure of ‘History of a mental health condition. Those who selected alcohol misuse or dependence were excluded from the alcohol BI analysis given that it is likely a confounder influencing the receipt of a BI for alcohol.

##### *Sociodemographic covariates*

Age was treated as a continuous variable in models, but categorical to summarise the sample characteristics. Other sociodemographic covariates included identified sex (Women vs other (Men and ‘In another way’/refused)), the presence of children in the household (Yes vs No), and region of England (North, Midlands and South).

### Outcome data collection periods

In the analyses of BIs for smoking, data were collected from March 2014 to June 2022. In the analyses of BIs for alcohol, data were collected from March 2014 to March 2022 because from April 2022 the brief intervention variable was collected every other month, and only questions related to AUDIT items one to three were collected (preventing the selection of individuals according to full 10-item AUDIT score).

For all primary analyses on BIs for smoking, and BIs for alcohol, the pre-pandemic period refers to the months up to and including February 2020, and the post-pandemic period from April 2020 onwards (no data were collected in March 2020 due to the pandemic). Characteristics of the sample for the pre- and post-pandemic periods are described in Table S1.

Regarding the analyses involving mental health data, the pre-pandemic period refers to the years 2016 and 2017, and the period from October 2020 onwards as the pandemic onset period, as these were the only periods where data on the included mental health measures were collected. Moreover, for 2016/2017 mental health was only assessed in past-year smokers, so this sample did not include any people who used alcohol at increasing and higher-risk levels but did not smoke.

### Analysis

The analyses were conducted in R version 4.2.1 [28] using the packages ‘survey’ [29] and ‘mgcv’ [30]. This analysis plan was pre-registered on the Open Science Framework 10.17605/OSF.IO/65FRC. The STROBE reporting guidelines were used in the design and reporting of this study. Respondents with missing data on any of the covariates of interest were excluded from the analyses (less than 5% of responses). Characteristics of the sample and descriptive statistics are presented using weighted descriptive statistics for the overall sample, and for the pre-pandemic and post-pandemic periods, respectively.

### Research question 1: was there a change in the receipt of BIs for smoking/alcohol between the pre-pandemic control period and the pandemic period, and did it depend on social grade?

A segmented regression design was used to assess the effect of the COVID-19 pandemic on receipt of BIs for smoking and alcohol, respectively. Data was analysed at the individual-level with segmented regression using generalised additive models (GAM) [31, 32]. These allow the fitting of seasonal smoothing terms and thus seasonality to be considered (which are particularly relevant in the context of delivery of interventions for smoking and alcohol use [33]). A log link function was used so that relative risks can be reported.

Each GAM modelled the trend in the *overall* receipt of BIs (dependent variable) for smoking and alcohol, respectively in the pre-pandemic period, and any change in the trends in the post-pandemic period. Trend is a variable coded 1…n (n being the total number of time-points to the end of the series) reflecting the time trend over time. The slope variable was defined as 0 before April 2020 of the pre-pandemic period and each month from April 2020, by increments of 1 up to *m* where *m* is the number of waves from April 2020.

Models were first fit assuming a linear underlying and post-implementation trend, followed by fits using non-linear trends to explore changes in the level of BI delivery and potential rebounding in the delivery of BIs over time. Specifically, the outcome of BI delivery refers to receipt of a BI in the previous 12 months. It is therefore possible that an immediate step change in delivery would not be detected in April 2020 or in the months immediately afterwards but would be reflected by changes in the trend in the longer term. In addition, after an initial drop during heightened restrictions 2020 and 2021, rates of BI delivery may have rebounded with some GP delivery returning to normal practice. Therefore, we fit further GAMs with the independent variables for slope and trend wrapped in a smooth function (model fit using the restricted maximum likelihood method with nine basis functions specified for the underlying trend and change in slope). Models accounted for seasonality in the receipt of BIs by using a smoothing term with cyclic cubic regression splines (11 knots, one for each month in the year) and were adjusted for sociodemographic characteristics (age, sex, children in the household, and region).

Interactions were tested between social grade and the post-intervention change in slope, and results reported stratified by social grade to explore whether the post-intervention slope depends on social grade. The model fit of the linear and non-linear GAMs were compared using the Akaike Information Criterion (AIC; lower values indicating better model fit) and a likelihood ratio test.

#### Sensitivity analyses

BI delivery may have declined during the pandemic due to reduced GP contact overall, rather than reduced delivery rates among those who visited their GP. To understand whether BI delivery also declined among those still visiting their GP, all analyses were repeated with the sample of only those who smoked in the past-year and those who used alcohol at increasing and higher risk levels, respectively, who reported visiting their GP in the past year (Table S2).

Models were checked for full convergence, and for randomly distributed residuals using the gam.check() function in the mgcv package [30] in R.

### Research question 2: was there a change in the receipt of BIs for smoking/alcohol between the pre-pandemic control period and the pandemic period, and did it depend on history of a mental health condition?

We constructed logistic regression models to explore whether changes in the of receipt of BIs for smoking and alcohol, respectively, from December 2016 to January 2017 and October 2020 to June (or March in the case of BIs for alcohol) 2022, depended on history of a mental health condition. Associations were reported as odds ratios (ORs) with 95% confidence intervals. Models adjusted for sociodemographic characteristics. The inclusion of the time period*mental health interaction allowed us to explore potentially differential changes in receipt of interventions over between the two time periods according to whether an individual had a history of a mental health condition.

## Results

A weighted total of 31,390 adults who smoked in the past year (mean (SD) age 41.9 (16.5), 46.4% women) and 22,386 who used alcohol at increasing and higher risk levels (AUDIT ≥ 8; (mean (SD) age 41.7 (16.4), 32.4% women)) completed the survey from March 2014 and June 2022 (March 2022 for sample who used alcohol at increasing and higher risk). Table 1 provides an overview of sample characteristics. The pre and post pandemic samples of those who smoked in the past year had broadly similar sociodemographic characteristics. The sample of those who used alcohol at increasing and higher risk levels included a lower proportion of 18–24 year-olds in the post-pandemic period.

**Table 1:** Sample characteristics (weighted) for the total study period (March 2014-June 2022), and before and after March 2020 in England for past-year smokers, and increasing and higher risk drinkers (AUDIT-C score of 4 or higher)

**Smoked in past year**	**Overall** *N* = 31,390		**Pre-March 2020** *N* = 23,119		**Post March 2020** *N* = 8,271	
**Characteristic**	%	95% CI	%	95% CI	%	95% CI
***Age***						
18–24	16.8	16.3, 17.2	16.7	16.2, 17.2	16.9	16.0, 17.9
25–34	24.1	23.6, 24.7	23.2	22.6, 23.8	26.8	25.7, 27.9
35–44	18.2	17.7, 18.7	18.3	17.7, 18.8	18.0	17.1, 19.0
45–54	17.4	17.0, 17.9	18.3	17.8, 18.9	14.9	14.1, 15.8
55–64	12.2	11.8, 12.6	12.6	12.3, 13.1	11.1	10.4, 11.8
65 +	11.2	10.9, 11.6	10.9	10.5, 11.3	12.2	11.5, 12.9
***Sex***						
Women	46.4	45.8, 47.0	46.6	45.9, 47.3	45.9	44.7, 47.1
***Social grade***						
ABC1	40.2	39.6, 40.8	39.1	38.4, 39.8	43.1	41.9, 44.3
C2DE	59.8	59.2, 60.4	60.9	60.2, 61.6	56.9	55.7, 58.1
***Region***						
London	14.4	14.0, 14.8	14.2	13.8, 14.7	14.9	14.0, 15.7
South	25.0	24.5, 25.6	24.4	23.8, 25.1	26.5	25.5, 27.6
Central	29.4	28.8, 29.9	29.2	28.6, 29.8	29.9	28.8, 31.1
North	31.2	30.7, 31.8	32.1	31.5, 32.8	28.7	27.6, 29.8
***Children in household***	32.7	32.1, 33.2	33.3	32.7, 34.0	30.7	29.6, 31.9
***BI for smoking***^a^	28.7	28.2, 29.3	30.5	29.9, 31.2	23.6	22.6, 24.7
***AUDIT 8 or higher***	**Overall** *N* = 22,386		**Pre March 2020** *N* = 15,842		**Post March 2020** *N* = 6,544	
***Age***						
18–24	20.9	20.4, 21.5	23.1	22.4, 23.8	15.7	14.8, 16.7
25–34	18.3	17.7, 18.9	17.8	17.1, 18.5	19.6	18.5, 20.7
35–44	17.3	16.7, 17.9	16.9	16.2, 17.6	18.3	17.2, 19.4
45–54	19.7	19.1, 20.3	19.3	18.6, 20.0	20.8	19.7, 21.8
55–64	13.7	13.3, 14.2	13.5	12.9, 14.0	14.4	13.6, 15.3
65 +	10.0	9.6, 10.4	9.5	9.0, 9.9	11.2	10.5, 12.0
***Sex***						
Women	32.4	31.8, 33.1	31.8	31.0, 32.6	33.9	32.7, 35.2
***Social grade***						
ABC1	61.1	60.4, 61.8	61.2	60.4, 62.0	60.8	59.5, 62.2
C2DE	38.9	38.2, 39.6	38.8	37.9, 39.6	39.2	37.8, 40.5
***Region***						
London	12.0	11.5, 12.4	11.0	10.5, 11.5	14.3	13.4, 15.2
South	26.8	26.1, 27.5	27.4	26.6, 28.2	25.4	24.3, 26.6
Central	23.9	23.3, 24.5	22.4	21.7, 23,1	27.5	26.3, 28.7
North	37.3	36.7, 38.0	39.2	38.4, 40.0	32.9	31.6, 34.1
***Children in household***	26.4	25.8, 27.1	25.2	24.4, 26.0	29.4	28.2, 30.7
***BI for alcohol***^**a**^	3.9	3.6, 4.1	3.8	3.5, 4.1	4.1	3.6, 4.6

Unweighted Ns Past-year smokers: Overall *N* = 30,438; Pre *N* = 23,119; Post *N* = 8,271

Unweighted Ns AUDIT ≥ 8 (used alcohol at increasing and higher risk levels): Overall *N* = 21,771; Pre *N* = 15,334; Post *N* = 6,437

^a^Among all adults including those who did not visit their GP

For the overall period, 28.7% (95% CI 28.2–29.3) of those who smoked in the past year received a BI for smoking cessation, but the prevalence of receipt of a BI declined from 30.5% (29.9–31.2) pre-pandemic to 23.6% (22.6–24.7) post-pandemic. Overall, 3.9% (3.6–4.7) of those who consumed alcohol at increasing and higher risk levels received a BI for alcohol, and the prevalence was similar both pre (3.8 (3.5–4.1)) and post (4.1 (3.6–4.6)) March 2020.

**Fig. 1:**
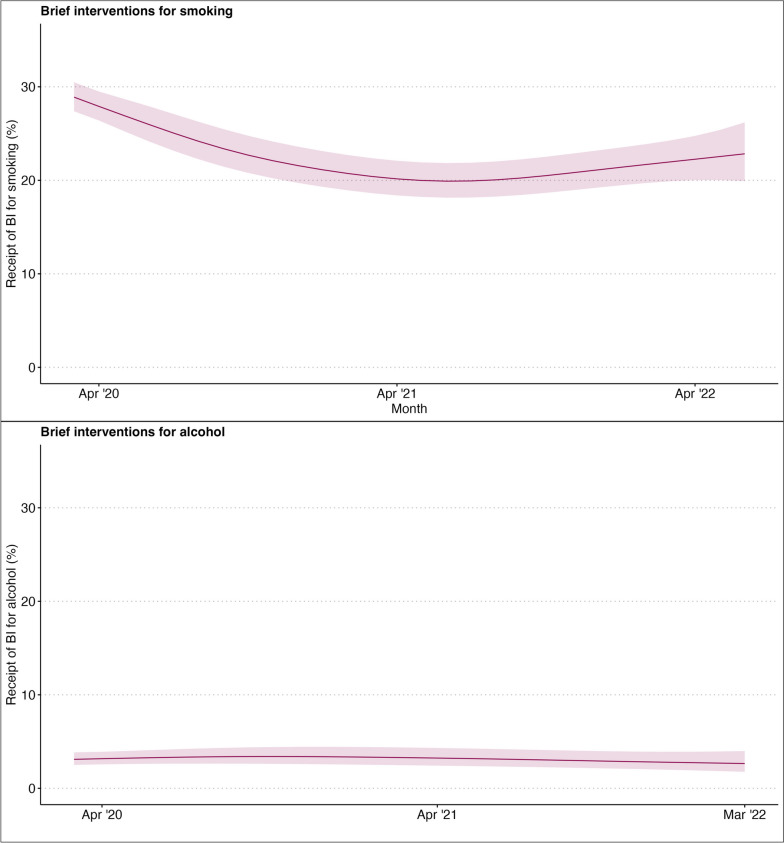
Predicted values for the percentage of past-year smokers, or increasing and higher risk alcohol users, reporting receipt of a brief intervention for smoking or alcohol – respectively—post-March 2020. *N* = 30,438. Lines represent predicted point estimates from the GAM modelled non-linearly using a smoothing term with nine basis functions. Shaded areas represent 95% confidence intervals

Results from the primary GAM analyses are presented in Table 2. Overall, the model including non-linear terms for the underlying trend and change in slope in BIs for smoking fit the data better than the linear model (χ ^2^(6) = 33.8, *p* < 0.001) (Table 2). In this non-linear model there was no decline in the receipt of BIs for smoking pre March 2020, but there was a change in the slope post March 2020. As illustrated in Fig. 1, the percentage of those who smoked reporting receipt of a BI after March 2020 declined steeply (from model predicted values of 27.9% (95% CI 26.4–29.5) in April 2020 and 19.9% (18.1–21.8) in June 2020).

**Table 2:** Results of the GAM analyses fitting non-linear trends to receipt of brief interventions for smoking, or alcohol use respectively

**Receipt of intervention for smoking**							
**Model 1 (linear trends)**	**RR**	**95% CI**	**z**	**edf**^**a**^	***p***	**AIC**	**LRT**^**b**^
Social grade C2DE (ref ABC1)	1.12	1.08, 1.16	6.10	-	< .001	32,505.24	(χ ^2^(6) = 33.1, *p* < .001)
Underlying trend	1.00	1.00, 1.00	-2.05	-	0.04		
Slope change	0.99	0.98, 0.99	-6.89		< .001		
**Model 2 (non-linear trends)**							
Social grade C2DE (ref ABC1)	1.12	1.08, 1.16	6.00	-	< .001	32,477.04	
Smooth term (underlying trend)	-	-	0.05	1.00	0.82		
Smooth term (slope change)	-	-	76.8	3.09	< .001		
**Receipt of intervention for alcohol (those with AUDIT 8 or higher**^**c**^**)**							
**Model 1 (linear trends)**	**RR**	**95% CI**	**z**	**edf**^**a**^	***p***	**AIC**	**LRT**^**b**^
Social grade C2DE (ref ABC1)	1.58	1.38, 1.81	6.59	-	< 0.001	6578.0	(χ ^2^(6) = 2.9, *p* < .02)
Underlying trend	1.00	1.00, 1.00	-0.38	-	0.70		
Slope change	0.99	0.98, 1.01	-0.67	-	0.50		
**Model 2 (non-linear trends)**							
Social grade C2DE (ref ABC1)	1.58	1.38, 1.81	6.59	-	< .001	6577.8	
Smooth term (underlying trend)	-	-	0.67	1.00	0.41		
Smooth term (slope change)	-	-	2.68	1.89	0.40		

^a^edf = effective degrees of freedom; all models adjusted for age, sex, children in the household and region

^b^Result of likelihood ratio test comparing model 1 with model 2

^c^Used alcohol at increasing and higher risk levels

**Fig. 2:**
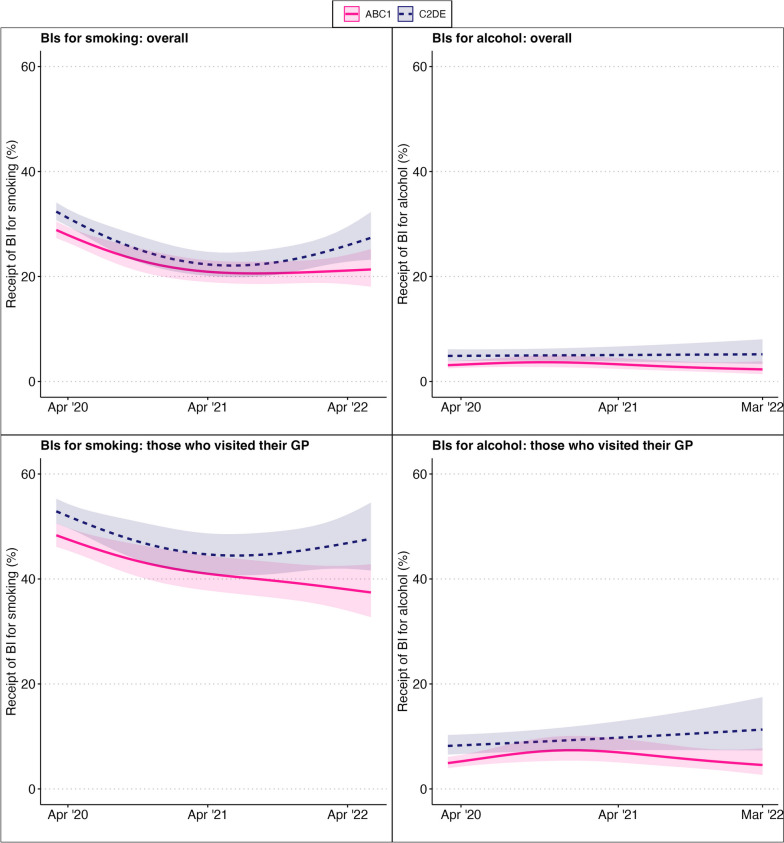
Percentage of past-year smokers or increasing and higher risk alcohol users reporting receipt of a brief intervention for smoking or alcohol—respectively—post-March 2020, overall and excluding those who did not visit their GP. BIs for smoking overall: *N* = 30,438; BIs for smoking: those who visited their GP: *N* = 19,300. BIs for alcohol overall: *N* = 21,771; BIs for alcohol: those who visited their GP: *N* = 14,146. Lines represent predicted point estimates from GAM allowing an interaction between slope and social grade, modelled non-linearly using a smoothing term with nine basis functions. Shaded areas represent 95% confidence intervals

In the non-linear model including an interaction term between the smooth terms for the change in slope and social grade (Table 3 and Fig. 2), there was an interaction such that the percentage of those who smoked reporting receipt of a BI after March 2020 declined steeply over the first year in both social grade ABC1 (model predicted values of 28.9% pre-March 2020, and 27.9% in month 1 to 20.1% in April 2021 post-pandemic) and C2DE (32.4% pre March 2020, and 31.1% in month 1 to 22.3% in month 13 post March 2020). After this point the trend stabilised at this lower level in ABC1 for the remainder of the period but rebounded among C2DE to 27.3% by the end of it. In the sensitivity analyses including only those who reported visiting their GP, the receipt of BIs for smoking declined from 48.1% (47.2–49.0; *N* = 14,679) pre-pandemic to 40.5% (39.0–42.0; *N* = 4,829) in the period post-pandemic. In the GAM analysis, the receipt of BIs post-March 2020 declined consistently in social grade ABC1 (model predicted values of 48.3% in month 1 to 37.4% at the end of the period), and appeared to level off after an initial decline in social grade C2DE (52.9% in month 1, 44.7% month 13, 47.7% in month 27) (Fig. 2 and Table 3).

**Table 3:** Results of the GAM analyses fitting linear and non-linear trends to receipt of brief interventions for smoking, or alcohol use, with an interaction between post-March 2020 slope and social grade, overall (Models 1 and 2) and excluding those who did not visit their GP in the past year (Models 3 and 4)

**Receipt of intervention for smoking**							
**Overall**							
**Model 1 (linear trends)**	**RR**	**95% CI**	**z**	**edf**^**a**^	***p***	**AIC**	**LRT**^**b**^
Social grade C2DE (ref ABC1)	1.12	1.07, 1.16	5.45	-	< .001	32,507.15	(χ ^2^(6) = 33.8, *p* < .001)
Underlying trend	1.00	1.00, 1.00	-2.06	-	0.04		
Slope change	0.99	0.98, 0.99	-5.76	-	< .001		
Slope x Social grade	1.00	1.00, 1.01	0.30	-	0.76		
**Model 2 (non-linear trends)**							
Social grade C2DE (ref ABC1)	1.12	1.08, 1.16	5.99	-	< .001	32,480.27	
Smooth term (underlying trend)	-	-	0.10	1.00	0.76		
Smooth term (slope change) x ABC1	-	-	48.53	2.43	< .001		
Smooth term (slope change) x C2DE	-	-	54.21	3.19	< .001		
**Excluding those who did not visit their GP in the past year**							
**Model 3 (linear trends)**	**RR**	**95% CI**	**z**	**edf**^**a**^	***p***	**AIC**	**LRT**^**b**^
Social grade C2DE (ref ABC1)	1.09	1.05, 1.13	4.93	-	< .001	23,565.39	(χ ^2^(6) = 9.04, *p* < .05)
Underlying trend	1.00	1.00, 1.00	-1.20	-	0.23		
Slope change	0.99	0.99, 0.99	-4.44	-	< .001		
Slope x Social grade	1.00	1.00, 1.01	0.30	-	0.76		
**Model 4 (non-linear trends)**							
Social grade C2DE (ref ABC1)	1.10	1.07, 1.14	5.98	-	< .001	23,561.58	
Smooth term (underlying trend)	-	-	0.14	1.01	0.71		
Smooth term (slope change) x ABC1	-	-	23.57	1.76	< .001		
Smooth term (slope change) x C2DE	-	-	15.93	2.11	< .001		
**Receipt of intervention for alcohol (those with AUDIT 8 or higher)**							
**Overall**							
**Model 1 (linear trends)**	**RR**	**95% CI**	**z**	**edf**^**a**^	***p***	**AIC**	**LRT**^**b**^
Social grade C2DE (ref ABC1)	1.54	1.32, 1.79	5.43	-	< 0.001	6579.40	(χ ^2^(6) = 36.2, *p* < .001)
Underlying trend	1.00	1.00, 1.00	-0.42	-	0.67		
Slope change	0.99	0.98, 1.01	-0.96	-	0.34		
Slope x Social grade	1.01	0.99, 1.03	0.79	-	0.43		
**Model 2 (non-linear trends)**							
Social grade C2DE (ref ABC1)	1.59	1.39, 1.82	6.66	-	< .001	6577.75	
Smooth term (underlying trend)	-	-	0.75	1.00	0.39		
Smooth term (slope change) x ABC1	-	-	4.99	2.06	0.20		
Smooth term (slope change) x C2DE	-	-	0.07	1.00	0.79		
**Excluding those who did not visit their GP in the past year**							
**Model 3 (linear trends)**	**RR**	**95% CI**	**z**	**edf**^**a**^	***p***	**AIC**	**LRT**^**b**^
Social grade C2DE (ref ABC1)	1.61	1.38, 1.88	6.12	-	< 0.001	5913.04	(χ ^2^(6) = 8.7, *p* < .05)
Underlying trend	1.00	1.00, 1.00	-0.72	-	0.47		
Slope change	1.01	0.99, 1.02	0.63	-	0.53		
Slope x Social grade	1.00	0.98, 1.02	0.42	-	0.68		
**Model 4 (non-linear trends)**							
Social grade C2DE (ref ABC1)	1.64	1.43, 1.87	7.20	-	< .001	5907.60	
Smooth term (underlying trend)	-	-	2.03	1.01	0.17		
Smooth term (slope change) x ABC1	-	-	8.58	2.24	0.04		
Smooth term (slope change) x C2DE	-	-	2.06	1.00	0.15		

^a^*edf* effective degrees of freedom; all models adjusted for age, sex, children in the household and region

^b^Result of likelihood ratio test comparing model 1 with model 2

There were no apparent linear or non-linear changes in the underlying trend or slope in the receipt of BIs for alcohol (Fig. 1 and Table 2). The receipt of BIs for alcohol among adults who used alcohol at increasing and higher risk levels that visited their GP was 5.8% (5.3–6.3; *N* = 3,313) pre-pandemic and 6.7% (5.9–7.6; *N* = 1,131) post-pandemic. As with the primary analysis, sensitivity analyses including only those who visited their GP showed no apparent linear or non-linear changes in receipt of BIs for alcohol (Fig. 2 and Table 3).

In the analyses examining changes in the receipt of BIs according to history of a mental health condition in 2016/2017 and or post-October 2020 (Table S3), the prevalence of those who smoked in the past year reporting a diagnosis with a mental health condition increased from 30.8% (30.1–32.2) in 2016/2017 to 44.2% (42.9–45.6) in the period from October 2020 to March 2022. There was no clear change in diagnosis with a mental health condition among those who used alcohol at increasing and higher risk levels between the periods.

In the logistic regression models examining the receipt of BIs for smoking, there was no interaction between time-period and history of a mental health condition (Table 4 and Figure S1). The interaction for drinking was unclear due to the small sample size (OR = 1.66, 95% CI 0.77–3.67; Figure S2). History of a mental health condition was associated with higher odds of receiving a BI for smoking (1.55, 95% CI 1.39–1.72) and possibly for alcohol (1.49, 0.73–3.02). Sensitivity analyses including only those who visited their GP showed a similar pattern of results (Tables S4 and S5).

**Table 4:** Association between receipt of a brief intervention for smoking or alcohol and ever diagnosis with a mental health condition^a^

**Receipt of brief intervention for smokinga**	**% (Total N)**	**OR**	**95% CI**	***p***
Time period				
2016/2017	32.1 (7,530)	-	-	
October 2020 onwards	23.0 (6,029)	0.61	0.54, 0.69	< 0.001
Ever diagnosis with MHC				
None	25.9 (8,549)	-	-	
Ever diagnosis	31.7 (5,010)	1.55	1.39, 1.72	< 0.001
Time period X ever diagnosis with MHC		0.98	0.84, 1.16	0.85
**Receipt of brief intervention for alcohola (those with *AUDIT 8 or higher)***				
Time period				
2016/2017	2.3 (1,485)	-	-	
October 2020 onwards	2.9 (4,528)	0.86	0.52, 1.48	0.56
Ever diagnosis with MHC				
None	2.1 (3,996)	-	-	
Ever diagnosis	4.1 (2,017)	1.49	0.73, 3.02	0.25
Time period X ever diagnosis with MHC		1.66	0.77, 3.67	0.20

Model adjusted for age, sex, social grade and region

^a^Among past year smokers, or those with AUDIT score of 8 or higher, respectively

## Discussion

In England, from March 2014 and February 2020 approximately one in three adults who smoked in the past year reported receiving a BI for smoking, and there was little change across this period. After March 2020, there was a change in the trend and after a 12-month decline the receipt of BIs settled at a lower level (approximately one in five adults who smoked in the past year) in social grade ABC1. In contrast, after a similar 12-month decline, the receipt of BIs for smoking among the less advantaged social grade C2DE (including those who are unemployed or in lower paid manual occupations) appeared to rebound and trend upwards until the end of the time period (June 2022). This is encouraging and reflects continued emphasis on targeting effective smoking cessation support at priority groups where the prevalence and harm from smoking is highest [3, 18]. There was no clear change in the receipt of BIs for alcohol across the period.

Regarding whether the delivery of BIs for smoking declined during the pandemic due to reduced GP contact overall, rather than reduced delivery rates among those who visited their GP, our sensitivity analyses that were restricted to only those who visited their GP showed followed a similar pattern to the primary analysis on the receipt of BIs for smoking. Therefore, the overall decline observed in the primary analysis cannot be fully explained by a drop in in-person patient contact during the pandemic. GPs were encouraged by health care regulatory bodies to use telemedicine where possible but reported little support or guidance in this transition and it is possible that this impacted their ability to conduct [34, 35]. While time-saving benefits of these remote consultations have been reported, concerns have been expressed among practitioners on their ability to connect and obtain adequate histories [34, 35], which should trigger a BI in the context of smoking. Future monitoring will indicate whether smoking BI delivery rates return to pre-pandemic levels and continue to prioritise those of less advantaged socioeconomic position.

Although those who used alcohol at increasing and higher risk levels from social grade C2DE were more likely to report receipt of a BI for alcohol than those in ABC1, the absolute rate of BI receipt was low (approximately 4% overall, and 6% in those who visited their GP) across the entire time period and seemingly unaffected by the COVID-19 pandemic. This is a continuation of the relatively low rate reported in analyses of data from the same source in 2014–2016 [3]. A recent systematic review of factors influencing the implementation of BIs for alcohol use in primary care highlighted several widespread barriers. In multiple studies, GPs and nurses reported a lack of time to address alcohol problems in the context of competing health issues, and limited training in how to address them. Moreover, where GPs were confident in their ability to screen and advise people who used alcohol, they were less confident in the impact it will have on their patients [36]. Other factors such as the attitudes and drinking behaviour of GPs themselves may influence the likelihood of BI delivery, wherein intervention delivery depends on the perception that a patient is at greater risk of harm from their consumption, or drinks more than them [37, 38]. Since the start of the pandemic, there has been a sustained rise in increasing and higher risk drinking [39], with a concurrent sharp rise in rates of alcohol-attributable mortality [40]. It appears that the relative rate of delivering BIs has not responded to this growing challenge.

Having a history of a mental health condition compared with none was associated with greater odds of receipt of a BI for smoking across the time periods studied. The fall in receipt of BIs for smoking over time did not depend on history of a mental health condition, with both groups experiencing a similar decline. Considering that the proportion reporting a history with a mental health condition has increased in recent years, the concurrent drop in BIs for smoking suggests that more could be done to support this priority group who suffer a disproportionate burden of smoking related morbidity and mortality [8]. There was no clear change in the receipt of BIs for alcohol across the period. The mental health analysis has two noteworthy limitations. First, the question on history of a mental health condition refers to “ever diagnosis” and individuals may not have been experiencing symptoms of a given mental health condition at the time of the survey. Second, because no data were collected between 2017 and 2020, the comparison of time periods is not sensitive to changes that might have occurred in the immediate years before the pandemic.

A limitation of the primary analysis is that the questions on receipt of BIs refer to receipt during the previous 12 months. Therefore, for up to a year from March 2020 the survey samples each month consisted of a decreasing proportion of respondents for whom the previous 12 months included the period of BI delivery before March 2020 (and a corresponding increasing proportion referring to the period after March 2020). Because of this, it is possible that the changes in the receipt of BIs for smoking reflect a step-level change, rather than the observed graduated decline. This is supported by the observation that when the sample only includes respondents who are referring to the post March 2020 period, there is an arrest in the decline. In other words, at this point the sample only includes those reflecting on the period of BI delivery at the lower level. Another limitation is that responses were self-reported (it is possible that for some individuals a response may not reflect their true smoking and alcohol use status, or receipt of a BI), and we do not have data on the content of BIs. Therefore, we cannot assess the fidelity of intervention delivery and how this may differ between groups or over time [37]. It is also not clear how potential declines in BIs for smoking have impacted population smoking cessation rates. Given the potentially adverse respiratory and cardiovascular morbidity outcomes caused by the interplay of COVID-19 and smoking [41], and the pressure on the NHS due to treatment of smoking-related morbidity, the context of the pandemic presented an opportunity for primary health care practitioners and public health professionals associated with the National Health Service to encourage smoking cessation through the launch of the GP initiated “Quit for COVID” social media campaign in the summer of 2020 [42]. In this campaign smokers were encouraged to quit and seek support from their local stop smoking service and available digital support for cessation. Quit rates increased following the pandemic [21] and it is possible that media campaigns such as “Quit for COVID” supported smoking cessation during the period of reduced BI delivery.

These results should be considered in the wider context of the resilience [43] of the health system in England. As described earlier, during the early stages of the pandemic, the health system had to adapt its functioning to cope with high numbers of seriously ill COVID-19 patients. As part of this, a proportion of in-person primary care shifted to remote digital or telephone consultation. In some areas of primary care such as sharing work between primary care specialists according to the nature of clinical need rather than who is available in clinic at a given time might have improved efficiency of diagnosis, treatment and referral [44]. Our results, supported by recent analyses using the same dataset [45], suggest that other components of primary care such as the delivery of BIs to support prevention of non-communicable diseases caused by smoking declined and have not returned to the same level of delivery compared with that during the pre-pandemic period. Should the UK government intensify policies to reduce cigarette smoking in ways it has recently outlined [46], services in the health system that support individuals from socioeconomically less advantaged groups, who generally have higher levels of cigarette dependence and are less likely to quit, remain essential to prevent a widening of smoking-related health inequalities.

## Conclusions

In conclusion, the receipt of BIs for smoking declined substantially after March 2020, coinciding with the onset of the COVID-19 pandemic and the associated social restrictions and changes in health care delivery. There is evidence that smoking BI rates rebounded among priority groups of people who smoked who are unemployed or in routine and manual occupations whereas amongst more advantaged groups declines in smoking BI delivery were maintained in the longer term. BIs for alcohol among those using alcohol at increasing and higher risk levels were higher among the same priority socioeconomic group compared with more advantaged people who used alcohol but remained low and stable throughout the surveyed period. Maintaining the higher BI delivery rates among priority groups – in terms of both socioeconomic and mental health status—of smokers and increasing and higher risk alcohol users is important to support reductions in smoking and alcohol related inequalities.

### Supplementary Information

**Additional file 1:** **Table S1**. Sociodemographic characteristics of those who visited their GP pre- and post-March 2020. **Table S2.** Sample characteristics for the total study period (March 2014-June 2022), and before and after March 2020 in England for past-year smokers, and increasing and higher risk drinkers (AUDIT score of 8 or higher), excluding those who did NOT visit their GP. **Table S3.** Sample characteristics for the total study period, and in 2016/2017 and or post-October 2020 in England for past-year smokers, and increasing and higher risk drinkers (AUDIT score of 8 or higher). **Table S4.** Sample characteristics for the total study period where mental health data was collected, 2016/2017 vs October 2020 onwards in England for past-year smokers, and increasing and higher risk drinkers (AUDIT score of 8 or higher), excluding those who did NOT visit their GP. **Table S5.** Association between receipt of a brief intervention for smoking or alcohol and ever diagnosis with a mental health condition* among those who visited their GP. **Figure S1. **Interaction plot for the receipt of brief intervention for smoking in 2016-2017 vs October 2020 onwards, by ever diagnosis with an MHC. **Figure S2. **Interaction plot for the receipt of brief intervention for alcohol in 2016-2017 vs October 2020 onwards, by ever diagnosis with an MHC.

## Data Availability

The pre-registered protocol and statistical code for this analysis is available at the open science framework 10.17605/OSF.IO/65FRC. Non-identifiable anonymised data used in the analysis are available—please contact the study author at l.kock@ucl.ac.uk.

## Abbreviations

BI: Brief intervention
GAM: Generalized additive model
GP: General practitioner
MHC: Mental health condition
SD: Standard deviation
STS/ATS: Smoking and Alcohol Toolkit Study
