# Supplementary material for: Brief interventions for smoking and alcohol associated with the COVID-19 pandemic: a population survey in England
Source: BMC Public Health. 2024 Jan 3;24:76. doi: 10.1186/s12889-023-17559-7 (PMC10763226; doi:10.1186/s12889-023-17559-7)
Supplement: Supplementary file 1 — Additional file 1: Table S1. Sociodemographic characteristics of those who visited their GP pre- and post-March 2020. Table S2. Sample characteristics for the total study period (March 2014-June 2022), and before and after March 2020 in England for past-year smokers, and increasing and higher risk drinkers (AUDIT score of 8 or higher), excluding those who did NOT visit their GP. Table S3. Sample characteristics for the total study period, and in 2016/2017 and or post-October 2020 in England for past-year smokers, and increasing and higher risk drinkers (AUDIT score of 8 or higher). Table S4. Sample characteristics for the total study period where mental health data was collected, 2016/2017 vs October 2020 onwards in England for past-year smokers, and increasing and higher risk drinkers (AUDIT score of 8 or higher), excluding those who did NOT visit their GP. Table S5. Association between receipt of a brief intervention for smoking or alcohol and ever diagnosis with a mental health condition* among those who visited their GP. Figure S1. Interaction plot for the receipt of brief intervention for smoking in 2016-2017 vs October 2020 onwards, by ever diagnosis with an MHC. Figure S2. Interaction plot for the receipt of brief intervention for alcohol in 2016-2017 vs October 2020 onwards, by ever diagnosis with an MHC. [file 12889_2023_17559_MOESM1_ESM.docx]

**SUPPLEMENTARY APPENDIX**

**Table S1**: Sociodemographic characteristics of those who visited their GP pre- and post-March 2020.

| **Smoked in past year** | **Pre-March 2020** N = 14,679 | | **Post March 2020** N = 4,829 | |
| --- | --- | --- | --- | --- |
| **Characteristic** | % | 95% CI | % | 95% CI |
| ***Age*** |  |  |  |  |
| 18-24 | 14.3 | 13.7, 14.9 | 17.5 | 16.3, 18.7 |
| 25-34 | 21.5 | 20.8, 22.3 | 27.0 | 25.6, 28.5 |
| 35-44 | 18.1 | 17.4, 18.8 | 17.7 | 16.5, 19.0 |
| 45-54 | 19.2 | 18.5, 19.9 | 14.4 | 13.4, 15.5 |
| 55-64 | 13.9 | 13.3, 14.5 | 10.9 | 10.0, 11.9 |
| 65+ | 13.1 | 12.6, 13.6 | 12.5 | 11.5, 13.4 |
| ***Sex*** |  |  |  |  |
| Women | 51.4 | 50.6, 52.3 | 51.3 | 49.8, 52.9 |
| ***Social grade*** |  |  |  |  |
| ABC1 | 38.7 | 37.9, 39.5 | 43.4 | 41.9, 45.0 |
| C2DE | 61.3 | 60.5, 62.1 | 56.6 | 55.0, 58.1 |
| ***Region*** |  |  |  |  |
| London | 14.1 | 13.5, 14.7 | 15.6 | 14.5, 16.8 |
| South | 24.7 | 23.9, 25.5 | 25.6 | 24.2, 27.0 |
| Central | 28.2 | 27.4, 29.0 | 29.9 | 28.4, 31.4 |
| North | 33.0 | 32.2, 33.8 | 28.9 | 27.5, 30.0 |
| ***Children in household*** | 33.7 | 32.9, 34.5 | 30.0 | 28.5, 31.4 |
| **AUDIT score 8 or higher** | **Pre March 2020** N = 3,313 | | **Post March 2020** N = 1,131 | |
| ***Age*** |  |  |  |  |
| 18-24 | 22.9 | 21.4, 24.4 | 25.9 | 23.1, 28.5 |
| 25-34 | 22.8 | 21.2, 24.4 | 28.2 | 25.3, 31.3 |
| 35-44 | 18.2 | 16.7, 19.8 | 19.5 | 16.9, 22.3 |
| 45-54 | 18,8 | 17.4, 20.4 | 12.9 | 10.9, 15.1 |
| 55-64 | 11.6 | 10.5, 12.7 | 8.4 | 6.9, 10.2 |
| 65+ | 5.7 | 5.0, 6.5 | 5.1 | 4.0, 6.5 |
| ***Sex*** |  |  |  |  |
| Women | 38,3 | 36.5, 40.1 | 42.4 | 39.3, 45.7 |
| ***Social grade*** |  |  |  |  |
| ABC1 | 48.5 | 46.7, 50.1 | 49.3 | 46.1, 52.6 |
| C2DE | 51.5 | 49.6, 53.3 | 50.7 | 47.4, 53.9 |
| ***Region*** |  |  |  |  |
| London | 11.3 | 10.3, 12.5 | 14.4 | 12.3, 16.7 |
| South | 25.5 | 23.8, 27.2 | 28.0 | 25.1, 31.1 |
| Central | 21.7 | 20.2. 23.3 | 26.9 | 24.1, 29.9 |
| North | 41.5 | 39.7, 43.4 | 30.7 | 27.8, 33.8 |
| ***Children in household*** | 27.3 | 25.6, 29.0 | 27.2 | 24.4, 30.2 |

**Table S2:** Sample characteristics for the total study period (March 2014-June 2022), and before and after March 2020 in England for past-year smokers, and increasing and higher risk drinkers (AUDIT score of 8 or higher), excluding those who did NOT visit their GP

| **Smoked in past year** | **Overall** N = 19,508 | | **Pre March 2020** N = 14,679 | | **Post March 2020**  N = 4,829 | | |  |
| --- | --- | --- | --- | --- | --- | --- | --- | --- |
| **Characteristic** | % | 95% CI | % | 95% CI | % | | 95% CI |  |
| ***Age*** |  |  |  |  |  | |  |  |
| 18-24 | 15.1 | 14.6, 15.6 | 14.3 | 13.7, 14.9 | | 17.5 | 16.3, 18.7 |  |
| 25-34 | 22.9 | 22.2, 23.6 | 21.5 | 20.8, 22.3 | | 27.0 | 25.6, 28.5 |  |
| 35-44 | 18.0 | 17.4, 18.6 | 18.1 | 17.4, 18.8 | | 17.7 | 16.5, 19.0 |  |
| 45-54 | 18.0 | 17.4, 18.6 | 19.2 | 18.5, 19.9 | | 14.4 | 13.4, 15.5 |  |
| 55-64 | 13.1 | 12.7, 13.6 | 13.9 | 13.4, 14.5 | | 10.9 | 10.0, 11.9 |  |
| 65+ | 12.9 | 12.5, 13.4 | 13.1 | 12.6, 13.6 | | 12.5 | 11.5, 13.4 |  |
| ***Sex*** |  |  |  |  | |  |  |  |
| Women | 51.4 | 50.6, 52.2 | 51.4 | 50.6, 52.3 | | 51.3 | 49.8, 52.9 |  |
| ***Social grade*** |  |  |  |  | |  |  |  |
| ABC1 | 39.9 | 39.1, 40.6 | 38.7 | 37.9, 39.5 | | 43.4 | 41.9, 45.0 |  |
| C2DE | 60.1 | 59.4, 60.9 | 61.3 | 60.5, 62.1 | | 56.6 | 55.0, 58.1 |  |
| ***Region*** |  |  |  |  | |  |  |  |
| London | 14.5 | 14.0, 15.0 | 14.1 | 13.5, 14.7 | | 15.6 | 14.5, 16.8 |  |
| South | 24.9 | 24.2, 25.6 | 24.7 | 23.9, 25.5 | | 25.6 | 24.2, 27.0 |  |
| Central | 28.6 | 27.9, 29.3 | 28.2 | 27.4, 29.0 | | 29.9 | 28.4, 31.4 |  |
| North | 32.0 | 31.3, 32.7 | 33.0 | 32.2, 33.8 | | 28.9 | 27.5, 30.3 |  |
| ***Children in household*** | 32.8 | 32.0, 33.5 | 33.7 | 32.9, 34.5 | | 30.0 | 28.5, 31.4 |  |
| ***Brief intervention for smoking**** | 46.2 | 45.5, 47.0 | 48.1 | 47.2, 49.0 | | 40.5 | 39.0, 42.0 |  |
| ***AUDIT score 8 or higher*** | **Overall** N = 14,335 | | **Pre March 2020** N = 10,367 | | | **Post March 2020** N = 3,968 | |  |
| ***Age*** |  |  |  |  | |  |  |  |
| 18-24 | 18.9 | 18.2, 19.6 | 20.1 | 19.3, 20.9 | | 14.8 | 14.6, 17.1 |  |
| 25-34 | 16.9 | 16.2, 17.6 | 16.2 | 15.4, 17.0 | | 18.9 | 17.5, 20.3 |  |
| 35-44 | 17.0 | 16.3, 17.7 | 17.0 | 16.2, 17.9 | | 17.1 | 15.8, 18.4 |  |
| 45-54 | 20.2 | 19.4, 20.9 | 20.1 | 19.2, 21.0 | | 20.4 | 19.1, 21.7 |  |
| 55-64 | 14.8 | 14.2, 15.4 | 14.8 | 14.1, 15.5 | | 14.8 | 13.7, 15.9 |  |
| 65+ | 12.2 | 11.7, 12.7 | 11.9 | 11.3, 12.5 | | 13.1 | 12.1, 14.2 |  |
| ***Sex*** |  |  |  |  | |  |  |  |
| Women | 36.2 | 35.3, 37.1 | 35.2 | 34.2, 36.2 | | 38.7 | 37.1, 40.3 |  |
| ***Social grade*** |  |  |  |  | |  |  |  |
| ABC1 | 62.3 | 61.4, 63.2 | 63.0 | 62.0, 64.1 | | 60.4 | 58.7, 62.1 |  |
| C2DE | 37.7 | 36.8, 38.6 | 37.0 | 35.9, 38.0 | | 39.6 | 37.9, 41.3 |  |
| ***Region*** |  |  |  |  | |  |  |  |
| London | 11.9 | 11.4, 12.4 | 10.9 | 10.3, 11.5 | | 14.6 | 13.4, 15.8 |  |
| South | 27.8 | 26.9, 28.6 | 28.6 | 27.6, 29.6 | | 25.6 | 24.1, 27.1 |  |
| Central | 23.4 | 22.7, 24.2 | 21.9 | 21.1, 22.8 | | 27.3 | 25.8, 28.8 |  |
| North | 36.9 | 36.0, 37.7 | 38.6 | 37.6, 39.6 | | 32.6 | 31.1, 34.2 |  |
| ***Children in household*** | 26.1 | 25.3, 26.9 | 25.2 | 24.3, 26.2 | | 28.4 | 26.9, 30.0 |  |
| ***Brief intervention for alcohol**** | 6.0 | 5.6, 6.5 | 5.8 | 5.3, 6.3 | | 6.7 | 5.9, 7.6 |  |

*Among all adults including those who did visit their GP
Unweighted Ns Past-year smokers: Overall N=19,300; Pre N=14,742; Post N=4,558
Unweighted Ns AUDIT ≥ 8 (used alcohol at increasing and higher risk levels): Overall N=14,146; Pre N=10,181; Post N=3,945

**Table S3:** Sample characteristics for the total study period, and in 2016/2017 and or post-October 2020 in England for past-year smokers, and increasing and higher risk drinkers (AUDIT score of 8 or higher)

| **Smoked in past year** | **Overall** N = 13,559 | | **2016/2017** N = 7,808 | | **October 2020 onwards**  N = 6,404 | |
| --- | --- | --- | --- | --- | --- | --- |
| **Characteristic** | % | 95% CI | % | 95% CI | % | 95% CI |
| ***Age*** |  |  |  |  |  |  |
| 18-24 | 17.0 | 16.4, 17.7 | 17.4 | 16.4, 18.2 | 16.8 | 15.8, 17.9 |
| 25-34 | 24.4 | 23.6, 25.2 | 22.7 | 21.6, 23.8 | 26.4 | 25.2, 27.7 |
| 35-44 | 18.1 | 17.4, 18.9 | 18.2 | 17.2, 19.2 | 18.0 | 17.0, 19.1 |
| 45-54 | 17.1 | 16.4, 17.8 | 18.6 | 17.7, 19.6 | 15.3 | 14.3, 16.3 |
| 55-64 | 11.8 | 11.2, 12.3 | 12.5 | 11.7, 13.2 | 11.0 | 10.2, 11.8 |
| 65+ | 11.6 | 11.1, 12.1 | 10.8 | 10.2, 11.5 | 12.5 | 11.7, 13.4 |
| ***Sex*** |  |  |  |  |  |  |
| Women | 46.5 | 45.6, 47.4 | 47.1 | 45.9, 48.3 | 45.8 | 44.4, 47.2 |
| ***Social grade*** |  |  |  |  |  |  |
| ABC1 | 41.8 | 40.9, 42.7 | 39.9 | 38.8, 41.1 | 44.1 | 42.7, 45.4 |
| C2DE | 58.2 | 57.3, 59.1 | 60.1 | 58.9, 61.2 | 55.9 | 54.6, 567.2 |
| ***Region*** |  |  |  |  |  |  |
| London | 14.2 | 13.6, 14.8 | 13.7 | 12.9, 14.5 | 14.8 | 13.8, 15.8 |
| South | 26.2 | 25.4, 27.0 | 25.6 | 24.6, 26.8 | 26.9 | 25.7, 28.1 |
| Central | 29.1 | 28.3, 29.9 | 28.7 | 27.7, 29.9 | 29.*5* | 28.2, 30.7 |
| North | 30.6 | 29.7, 31.4 | 31.9 | 30.8, 33.0 | 28.9 | 27.7, 30.2 |
| ***Children in household*** | 32.0 | 31.1, 32.9 | 33.8 | 32.7, 35.0 | 29.8 | 28.5, 31.1 |
| ***Brief intervention for smoking**** | 27.0 | 26.2, 27.8 | 30.8 | 29.7, 31.9 | 22.4 | 21.2, 23.5 |
| ***Ever diagnosis with MHC*** | 37.1 | 36.2, 38.0 | 30.8 | 30.1, 32.4 | 44.2 | 42.9, 45.6 |
| ***AUDIT 8 or higher*** | **Overall** N = 6,165 | | **2016/2017** N = 1,588 | | **October 2020 onwards** N = 4,578 | |
| ***Age*** |  |  |  |  |  |  |
| 18-24 | 19.6 | 18.6, 20.7 | 30.2 | 27.9, 32.7 | 16.0 | 14.8, 17.2 |
| 25-34 | 20.6 | 19.4, 21.8 | 23.4 | 21.1, 26.0 | 19.6 | 18.3, 20.9 |
| 35-44 | 17.7 | 16.6, 18.8 | 15.7 | 13.7, 17.9 | 18.4 | 17,1, 19.7 |
| 45-54 | 19.8 | 18.8, 19.9 | 16.9 | 15.0, 19.1 | 20.8 | 19.6, 22.1 |
| 55-64 | 13.3 | 12.4, 14.1 | 9.9 | 8.6, 11.5 | 14.4 | 13.4, 15.5 |
| 65+ | 9.1 | 8.4, 9.8 | 3.8 | 3.0. 4.8 | 10.9 | 10.0, 11.8 |
| ***Sex*** |  |  |  |  |  |  |
| Women | 34.7 | 33.4, 36.0 | 36.9 | 34.4, 39.5 | 34.0 | 32.5, 35.4 |
| ***Social grade*** |  |  |  |  |  |  |
| ABC1 | 59.1 | 57.7, 60.4 | 50.4 | 47.7, 53.2 | 62.0 | 60.4, 63.6 |
| C2DE | 40.9 | 39.6, 42.3 | 49.6 | 46.8, 52.3 | 38.0 | 36.4, 39.6 |
| ***Region*** |  |  |  |  |  |  |
| London | 13.1 | 12.2, 14.0 | 10.2 | 8.7, 11.8 | 14.1 | 13.1, 15.2 |
| South | 25.7 | 24.5, 27.0 | 26.0 | 23.6, 28.6 | 25.6 | 24.3, 27.1 |
| Central | 26.2 | 25.0, 27.4 | 22.6 | 20.3, 24.9 | 27.5 | 26.1, 28.9 |
| North | 34.9 | 33.7, 36.2 | 41.3 | 38.7, 44.0 | 32.7 | 31.2, 34.2 |
| ***Children in household*** | 28.4 | 27.1, 29.6 | 25.8 | 23.5, 28.4 | 29.2 | 27.8, 30.7 |
| ***Brief intervention for alcohol**** | 2.7 | 2.3, 3.2 | 2.3 | 1.6, 3.2 | 2.8 | 2.4, 3.4 |
| ***Ever diagnosis with MHC*** | 33.7 | 32.4, 35.0 | 34.4 | 31.9, 37.0 | 33.4 | 31.9, 34.9 |

*Among all adults including those who did visit their GP; Mental Health Condition (MHC)
Unweighted Ns Past-year smokers: Overall N=13,559; Pre N=7,530; Post N=6,029
Unweighted Ns AUDIT ≥ 8: Overall N=6,013; Pre N=1,485; Post N=4,528

**Table S4:** Sample characteristics for the total study period where mental health data was collected, 2016/2017 vs October 2020 onwards in England for past-year smokers, and increasing and higher risk drinkers (AUDIT score of 8 or higher), excluding those who did NOT visit their GP

| **Smoked in past year** | **Overall** N = 8,658 | | **2016/2017** N = 5,031 | | **October 2020 onwards**  N = 3,627 | |
| --- | --- | --- | --- | --- | --- | --- |
| **Characteristic** | % | 95% CI | % | 95% CI | % | 95% CI |
| ***Age*** |  |  |  |  |  |  |
| 18-24 | 16.3 | 15.5, 17.2 | 15.3 | 14.3, 16.4 | 17.6 | 16.3, 19.1 |
| 25-34 | 23.7 | 22.7, 24.7 | 21.4 | 20.1, 22.6 | 26.9 | 25.2, 28.5 |
| 35-44 | 17.7 | 16.8, 18.6 | 17.6 | 16.5, 18.8 | 17.8 | 16.4, 19.3 |
| 45-54 | 17.2 | 16.4, 18.1 | 19.0 | 17.9 20.2 | 14.8 | 13.6, 16.0 |
| 55-64 | 12.3 | 11.7, 13.4 | 13.6 | 12.7, 14.5 | 10.6 | 9.6, 11.7 |
| 65+ | 12.3 | 12.1, 13.4 | 13.1 | 12.2, 14.0 | 12.3 | 11.2, 13.4 |
| ***Sex*** |  |  |  |  |  |  |
| Women | 51.5 | 50.4, 52.7 | 51.8 | 50.3, 53.3 | 51.1 | 49.2, 52.9 |
| ***Social grade*** |  |  |  |  |  |  |
| ABC1 | 41.5 | 40.4, 42.6 | 39.6 | 38.2, 41.0 | 44.1 | 42.4, 45.9 |
| C2DE | 58.5 | 57.4, 59.6 | 60.4 | 59.0, 61.8 | 55.9 | 54.1, 57.6 |
| ***Region*** |  |  |  |  |  |  |
| London | 14.8 | 14.0, 15.6 | 14.2 | 13.2, 15.2 | 15.5 | 14.3, 16.9 |
| South | 25.8 | 24.7, 26.8 | 25.7 | 24.3, 27.1 | 25.9 | 24.3, 27.5 |
| Central | 28.3 | 27.3, 29.4 | 27.9 | 26.6, 29.2 | 29.0 | 27.4, 30.7 |
| North | 31.1 | 30.1, 32.2 | 32.2 | 30.9, 33.6 | 29.6 | 28.0, 31.3 |
| ***Children in household*** | 31.6 | 30.5, 32.7 | 33.2 | 31.8, 34.6 | 29.4 | 27.7, 31.1 |
| ***Brief intervention for smoking**** | 44.3 | 43.2, 45.5 | 47.8 | 46.3, 49.3 | 39.5 | 37.7, 41.3 |
| ***Ever diagnosis with MHC*** | 42.2 | 41.0, 43.3 | 36.2 | 34.8, 37.6 | 50.5 | 48.7, 52.3 |
| ***AUDIT 8 or higher*** | **Overall** N = 3,614 | | **2016/2017** N = 994 | | **October 2020 onwards** N = 2,620 | |
| ***Age*** |  |  |  |  |  |  |
| 18-24 | 20.3 | 18.9, 21.7 | 30.8 | 27,8, 34.0 | 16.3 | 14.8, 17,9 |
| 25-34 | 20.3 | 18.9, 21,8 | 22.8 | 19.9, 25.9 | 19.4 | 17.7, 21.2 |
| 35-44 | 16.2 | 14.9, 17.6 | 14.2 | 12.0, 16.9 | 16.9 | 15.3, 18.6 |
| 45-54 | 19.3 | 17.9, 20.7 | 16.7 | 14.3, 19.4 | 20.3 | 18.7, 21.9 |
| 55-64 | 13.6 | 12.5, 14.8 | 10.9 | 9.1, 13.0 | 14.7 | 13.3, 16.1 |
| 65+ | 10.3 | 9.4, 11.4 | 4.6 | 3.5, 5.9 | 12.5 | 11.3, 13.9 |
| ***Sex*** |  |  |  |  |  |  |
| Women | 39.8 | 38.1, 41.5 | 42.7 | 39.4, 46.0 | 38.7 | 36.7, 40.7 |
| ***Social grade*** |  |  |  |  |  |  |
| ABC1 | 58.7 | 56.9, 60.4 | 50.6 | 47.2, 53.9 | 61.8 | 59.6, 63.8 |
| C2DE | 41.3 | 39.5, 43.1 | 49.4 | 46.1, 52.8 | 38.2 | 36.2, 40.4 |
| ***Region*** |  |  |  |  |  |  |
| London | 13.4 | 12.3, 14.6 | 10.2 | 8.4, 12.3 | 14.6 | 13.2, 16.1 |
| South | 26,3 | 24.7, 27.9 | 26.9 | 23.8, 30.1 | 26.1 | 24.3, 28.0 |
| Central | 26.1 | 24.6, 27.7 | 22.8 | 20.1, 25.8 | 27.4 | 25.6, 29.2 |
| North | 34.2 | 32.5, 35.9 | 40.2 | 36.9, 43.4 | 31.9 | 30.0, 33.9 |
| ***Children in household*** | 27,6 | 26.0, 29.2 | 25.1 | 22.2, 28.2 | 28,5 | 26.6, 30.0 |
| ***Brief intervention for alcohol**** | 4.6 | 3.9, 5.4 | 3.6 | 2.5, 5.1 | 5.0 | 4.1, 5.9 |
| ***Ever diagnosis with MHC*** | 39.7 | 38.0, 41.4 | 40.4 | 37.2, 43.8 | 39.4 | 37.4, 41.5 |

*Among those who did visit their GP
*Among all adults including those who did visit their GP; Mental Health Condition (MHC)
Unweighted Ns Past-year smokers: Overall N=8,450; Pre N=4,976; Post N=3,474
Unweighted Ns AUDIT ≥ 8: Overall N=3,562; Pre N=954; Post N=2,608

**Table S5:** Association between receipt of a brief intervention for smoking or alcohol and ever diagnosis with a mental health condition* among those who visited their GP

| **Receipt of brief intervention for smoking*** | **% (Total N)** | **OR** | **95% CI** | ***p*** |
| --- | --- | --- | --- | --- |
| Time period |  |  |  |  |
| 2016/2017 | 48.6 (4,976) | - | - |  |
| October 2020 onwards | 40.0 (3,474) | 0.76 | 0.67, 0.85 | <0.001 |
| Ever diagnosis with MHC |  |  |  |  |
| None | 45.0 (4,923) | - | - |  |
| Ever diagnosis | 45.1 (3,527) | 1.20 | 1.06, 1.35 | 0.003 |
| Time period X ever diagnosis with MHC | 40.0 (1.758) | 0.93 | 0.77, 1.11 | 0.41 |
| **Receipt of brief intervention for alcohol*** |  |  |  |  |
| Time period |  |  |  |  |
| 2016/2017 | 3.6 (954) | - | - |  |
| October 2020 onwards | 5.1 (2,608) | 0.99 | 0.60, 1.72 | 0.98 |
| Ever diagnosis with MHC |  |  |  |  |
| None | 3.9 (2,157) | - | - |  |
| Ever diagnosis | 5.8 (1,405) | 1.23 | 0.59, 2.47 | 0.57 |
| Time period X ever diagnosis with MHC | 4.5 (1,017) | 1.67 | 0.77, 3.70 | 0.20 |

Model adjusted for age, sex, social grade and region; *Among past year smokers, or those with AUDIT score of 8 or higher, respectively who visited their GP

**Figure S1:** Interaction plot for the receipt of brief intervention for smoking in 2016-2017 vs October 2020 onwards, by ever diagnosis with an MHC

**
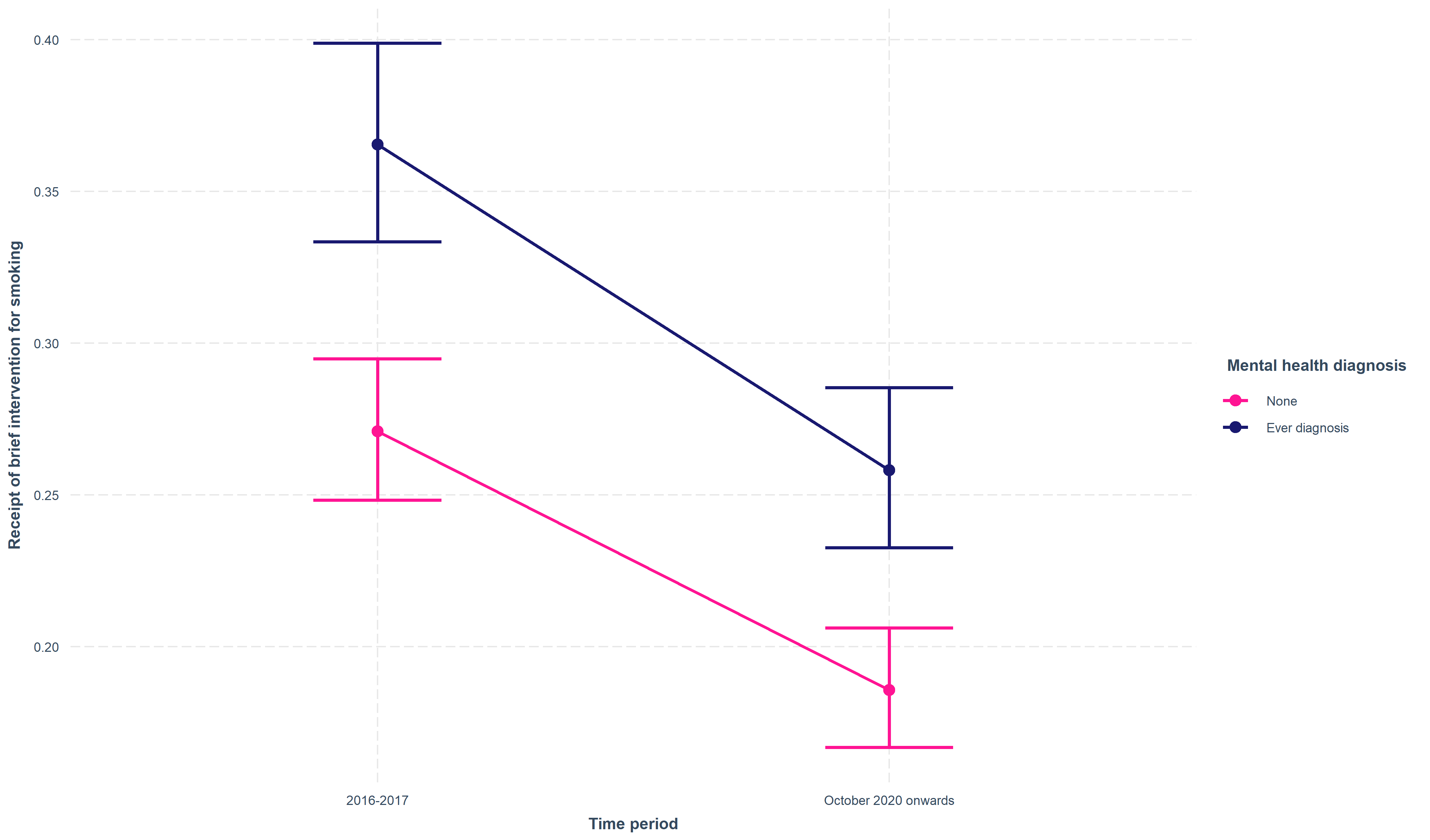
**

**Figure S2:** Interaction plot for the receipt of brief intervention for alcohol in 2016-2017 vs October 2020 onwards, by ever diagnosis with an MHC

**
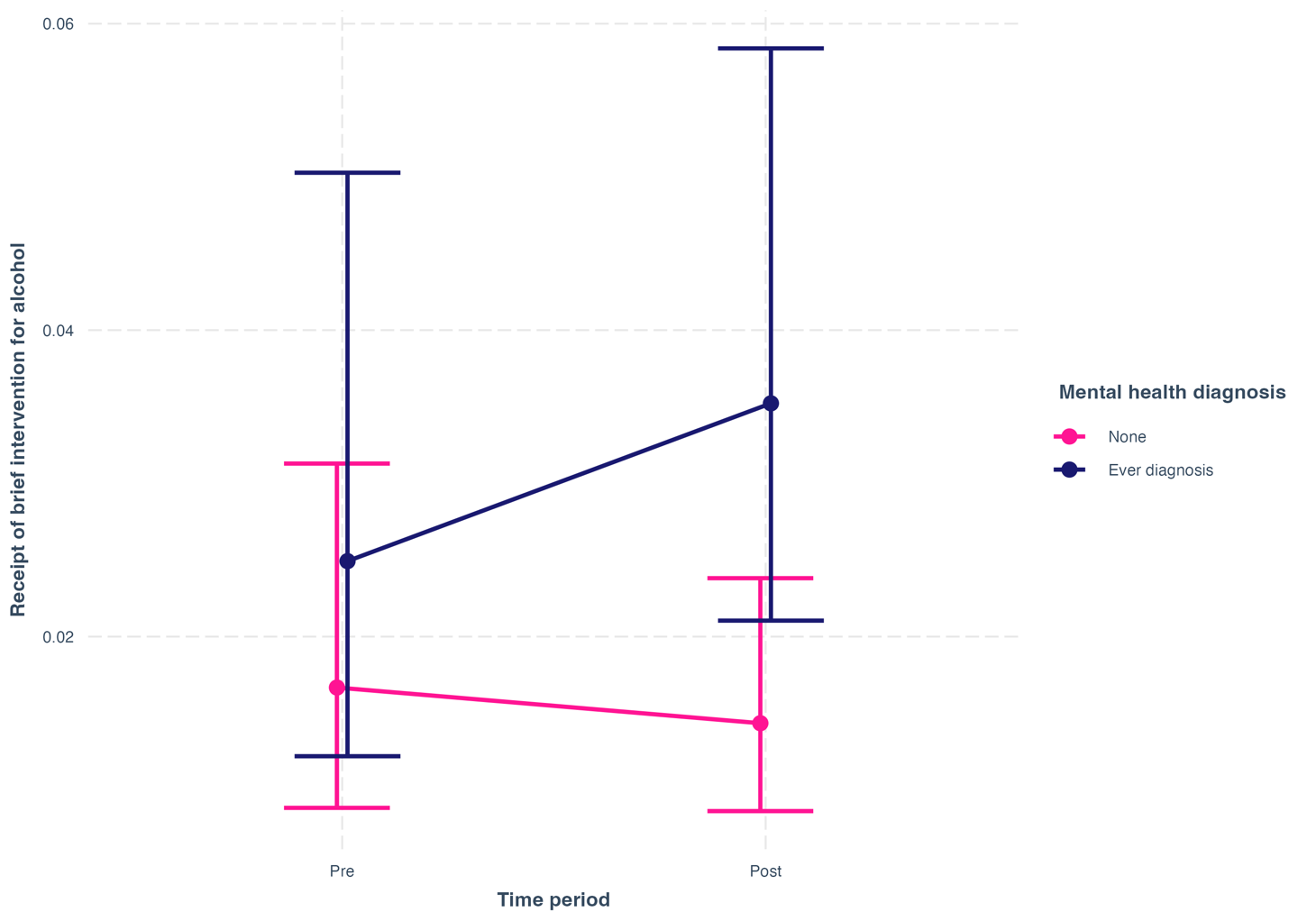
**
